# Environmental Factors Associated with the Distribution of *Anopheles gambiae* s.s in Ghana; an Important Vector of Lymphatic Filariasis and Malaria

**DOI:** 10.1371/journal.pone.0009927

**Published:** 2010-03-29

**Authors:** Dziedzom de Souza, Louise Kelly-Hope, Bernard Lawson, Michael Wilson, Daniel Boakye

**Affiliations:** 1 Noguchi Memorial Institute for Medical Research, University of Ghana, Legon-Accra, Ghana; 2 Department of Theoretical and Applied Biology, Kwame Nkrumah University of Science and Technology, Kumasi, Ghana; 3 Centre for Neglected Tropical Diseases, University of Liverpool, Liverpool, United Kingdom; New York University, United States of America

## Abstract

*Anopheles gambiae s.s* mosquitoes are important vectors of lymphatic filariasis (LF) and malaria in Ghana. To better understand their ecological aspects and influence on disease transmission, we examined the spatial distribution of the *An. gambiae* (M and S) molecular forms and associated environmental factors, and determined their relationship with disease prevalence. Published and current data available on the *An. gambiae* species in Ghana were collected in a database for analysis, and the study sites were georeferenced and mapped. Using the *An. gambiae s.s* sites, environmental data were derived from climate, vegetation and remote-sensed satellite sources, and disease prevalence data from existing LF and malaria maps in the literature. The data showed that *An. gambiae* M and S forms were sympatric in most locations. However, the S form predominated in the central region, while the M form predominated in the northern and coastal savanna regions. Bivariate and multiple regression analyses identified temperature as a key factor distinguishing their distributions. *An. gambiae* M was significantly correlated with LF, and 2.5 to 3 times more prevalent in the high LF zone than low to medium zones. There were no significant associations between high prevalence *An. gambiae* s.s locations and malaria. The distribution of the *An. gambiae* M and S forms and the diseases they transmit in Ghana appear to be distinct, driven by different environmental factors. This study provides useful baseline information for disease control, and future work on the *An. gambiae* s.s in Ghana.

## Introduction

The mosquito *Anopheles gambiae* sensu lato (s.l.) contains seven species, of which *An. gambiae sensu stricto* (s.s), *An. arabiensis* and *An. melas* are three of the major vectors of lymphatic filariasis (LF) and malaria caused by *Wuchereria bancrofti*, and *Plasmodium falciparum* respectively in West Africa [1], [2]. In Ghana, previous studies have found the *An. gambiae* s.l and the *An. funestus* to be the major vectors of LF in the southern coastal zone and in the northern region of the country [3]–[7].

Vector control is considered an important tool for diseases transmitted by mosquitoes and other insect vectors [8]. The current Ghana policy on vector control against *Anopheles* vectors prioritizes the use of insecticide treated materials and indoor residual spraying. However, the efficiency of these interventions will depend on information on the distribution and abundance of the main vectors, the specific molecular forms of *An. gambiae* s.s and the levels of insecticide resistance within them [9], [10].

Studies on the distribution of the *An. gambiae* complex in Africa [9], [11], highlighted gaps in our knowledge, and Coetzee and colleagues [9] further recommended an urgent need for baseline surveys on the distribution of these malaria vectors in areas where no reliable information exists. Ghana is one of these countries. The largest and most recent study published on the abundance, distribution and levels of insecticide resistance in *An. gambiae* s.s covered 11 sites across different ecological zones of the country [12]. This study found that the *An. gambiae* S form predominated across the country, except in the arid north where only the M form was found. While this was the first major attempt at presenting the geographical distribution and ecological variations in *An. gambiae* s.s in Ghana, it is not known how these data compare with other *An. gambiae* s.s data, whether there are specific environmental factors driving the distributions of the M and S molecular forms, and if there are any associations with LF and malaria distributions [13], [14].

In order to better understand the ecological aspects of this important vector, their influence on the epidemiology of LF and malaria, this study collated data on *An. gambiae* s.s in Ghana, and aimed to i) examine the spatial distribution of the *An. gambiae* M and S forms across the country, ii) identify key environmental factors associated with their distribution, and iii) determine their relationship with LF and malaria prevalence distributions. It is envisaged that this information will help develop a comprehensive profile on the ecology of *An. gambiae* s.s in Ghana, which will assist researchers and the diseases control programs in the country.

## Methods

### Study site

The Republic of Ghana is a developing West African country bordered to the north by Burkina Faso, to the east by Togo, to the West by Cote d'Ivoire and to the south by the Gulf of Guinea [15]. It has a total surface area of 239, 460 sq km with a coastline of 539 km, and approximately a population of 23 million inhabitants. The climate is warm and comparatively dry along the southeast coast, hot and humid in the southwest, and hot and dry in north. The ecology can be divided into six zones; the mangrove zones at the coastline, the coastal savanna, the evergreen forest in the south-west, the moist semi-deciduous forest in the central area, the guinea savanna in the north and the sudan savanna in the north-east. The terrain is mostly low plain, with dissected plateau in the south-central area. The elevation ranges from 0 to 880 m above sea level. There are two main seasons; a rainy season from April to October and dry season from November to March.

### Entomological data and mapping

Entomological studies on *An. gambiae* s.l in Ghana were identified from various sources including published articles in peer-reviewed journals, unpublished works from MPhil and PhD theses held at the Noguchi Memorial Institute for Medical Research (NMIMR), Accra-Ghana, as well as on-going studies at NMIMR. The collated data spanned from 2001 to 2008. Information on the location, study period, sample size, collection method, mosquito species and molecular forms, from each study were collated into a database. Various collection methods including; human landing catches, pyrethroid spray catches, larval collections and aspirators were used, depending on the location of the study sites and the objectives of the various studies. However, irrespective of the collection method, data collected from the same location in different years were considered separately. With the exception of few sites, most locations (i.e collection site) in the database were geo-referenced using the latitude and longitude coordinates obtained by cross-checking the names with data from the GEOnet Names Server [16], and Directory of Cities and Towns in the World [17] databases.

All data were imported into the geographical information systems software ArcGIS 9.2 (ESRI, Redlands, CA) for mapping and spatial analyses. First, the overall distribution of the *An. gambiae* s.l, and the different prevalence distributions of *An. gambiae* M and S across the country were mapped. Mosquito collection methods were also compared, to highlight differences in sampling. Second, spatial analysis of the *An. gambiae* M and S prevalences were examined using ArcGIS Spatial Analyst and Statistics tools (ESRI, Redland, CA). The Moran's *I* statistic was used to determine spatial autocorrelation patterns i.e. clustered, dispersed, random, and the Getis-Ord Gi* statistic was used to identify the specific locations where high and low prevalences were clustered (Z scores, 95% confidence levels (CI) +1.96 and −1.96 standard deviations). In addition, the kernel density estimation (KDE) method, non-parametric way of estimating the probability density function, was used to create a continuous surface representing the high to low density distributions of each molecular form.

### Environmental data and analysis

To examine environmental factors associated with the prevalence distributions of *An. gambiae* M and S in Ghana, specific data on elevation, vegetation, precipitation, temperature and humidity were obtained for each location (i.e. collection site), and compiled into a database, for descriptive and statistical analyses in SPSS 16.0 (SPSS, Inc, Chicago, IL).

Elevation data were derived from the U.S Geological Survey's ETOPO2 Digital Elevation Model available from ESRI (Redlands, CA). The elevation at each collection site was determined by importing the digital elevation map into ArcGIS 9.2 and extracting the underlying value (metres). Vegetation and climate data were based on the mean annual values for the specific year of study, obtained from the best available sources via the Climate Data Library of the International Research Institute for Climate and Society [18]. Vegetation cover was based on Normalized Difference Vegetation Index (NDVI) satellite data extracted from the LandDAAC MODIS version 005 West Africa from USGS [19], [20]. Precipitation (mm), temperature (C°) and specific humidity (qa) measures were obtained from satellite data from the National Oceanic and Atmospheric Administration (NOAA) and based on daily mean readings taken 2 meters above the ground [21]–[25].

First, the relationship between *An. gambiae* M and S, and each environmental variable was examined using bivariate correlations, Pearson's correlation coefficient (2 tailed *P* values≤0.05 significance). Stepwise multiple linear regression analysis was then used to identify the environmental factor that would best predict the distribution of each molecular form. To account for environmental variables that may be highly correlated with each other, the level of colinearity tolerance in the stepwise regression procedure was set at ≥0.8 and only variables above this threshold were accepted in the models. Second, to better understand the environmental parameters associated with the *An. gambiae* M and S forms, mean environmental measures between high and low prevalence sites were compared using the Mann-Whitney *U* test with Bonferroni correction for multiple comparisons.

### Relationship with disease prevalence distributions

To examine the relationship between the *An. gambiae* M and S prevalence and the distribution of disease, maps on the LF prevalence [13] and *P. falciparum* prevalence [14] for West Africa were imported into ArcGIS 9.2 and geo-referenced. The LF map was modeled from the *W. bancrofti* seroprevalence data collected in 2000 from 401 villages throughout Benin, Burkina Faso, Ghana and Togo [13]. The *P. falciparum* malaria prevalence map was modelled on extensive data obtained from children aged 2–10 years in non-epidemic periods, using a generalized linear mixed model [14].

The *An. gambiae* s.s collection sites were used as focal points, whereby the underlying disease prevalence data could be compared with the entomological data. The LF and malaria prevalence data, corresponding to the latitude and longitude of each mosquito collection site, were extracted and exported for descriptive and statistical analyses, which included bivariate correlations, and comparison of means between high and low prevalence sites of the *An. gambiae* M and S forms.

Further, it was of particular interest to explore the entomological and environmental characteristics in different LF transmission zones based on the prevalence data map in Gyapong et al. 2002 [13]. The prevalence distributions ranged from 0 to 30%, and were classified into distinct transmission zones, which were digitized in ArcGIS 9.2. The entomological and environmental data from each of collection sites within each zone were summarized, and significant differences identified by comparing the standard errors (+2SE) of the means.

## Results

### Entomological mapping

**Figure 1:**
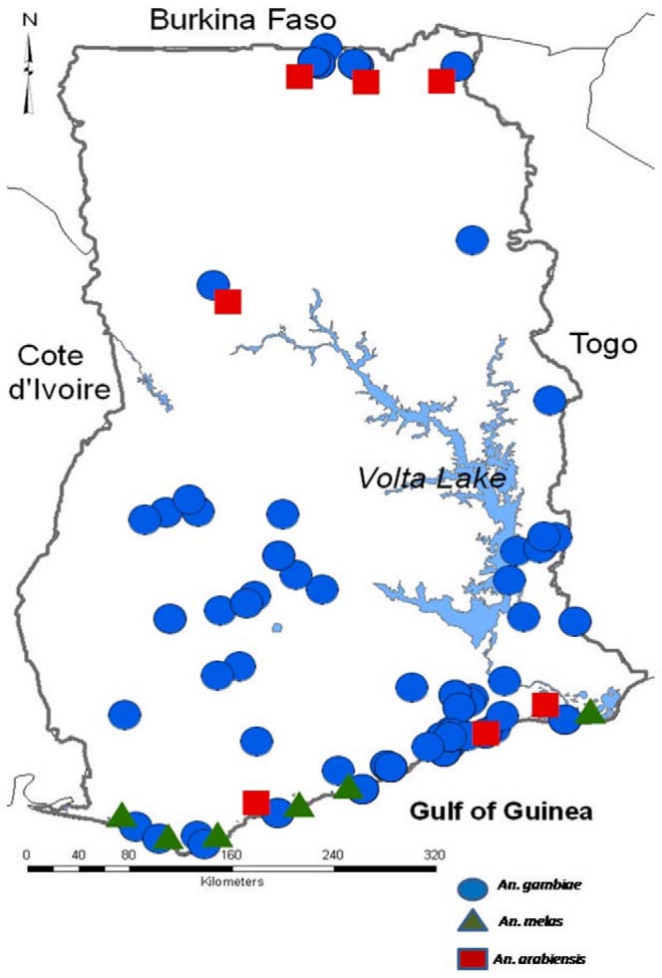
Distribution of *An. gambiae* s.l mosquitoes.

The collated *An. gambiae* species complex database contained 143 records with a total of 12,607 mosquitoes, reflecting both larval and adult catches. From this, the distribution of *An. gambiae* s.l was mapped (Figure 1). The most dominant species was *An. gambiae* s.s, which was found at 114 sites (total n = 10,028), followed by *An. melas* (6 sites, total n = 469) and *An. arabiensis* (8 sites, total n = 240). The map indicates that the distribution of *An. gambiae* s.s was widespread, while *An. melas* was primarily found along the coastal Savanna zones, a predominantly marshy environment, and *An. arabiensis* mainly in the northern savanna zone. *Anopheles funestus*, the second most important vector of malaria and lymphatic filariasis in Ghana, was also recorded in 9 sites with a total of 1,825 mosquitoes. Refer to supplementary data File S1 “*Anopheles* distribution records in Ghana” for further information on *An. funestus* and other *Anopheles* mosquito species, collection sites and coordinates, year and month of collection (where available), collection methods, numbers collected and identified, and the data sources.

**Figure 2:**
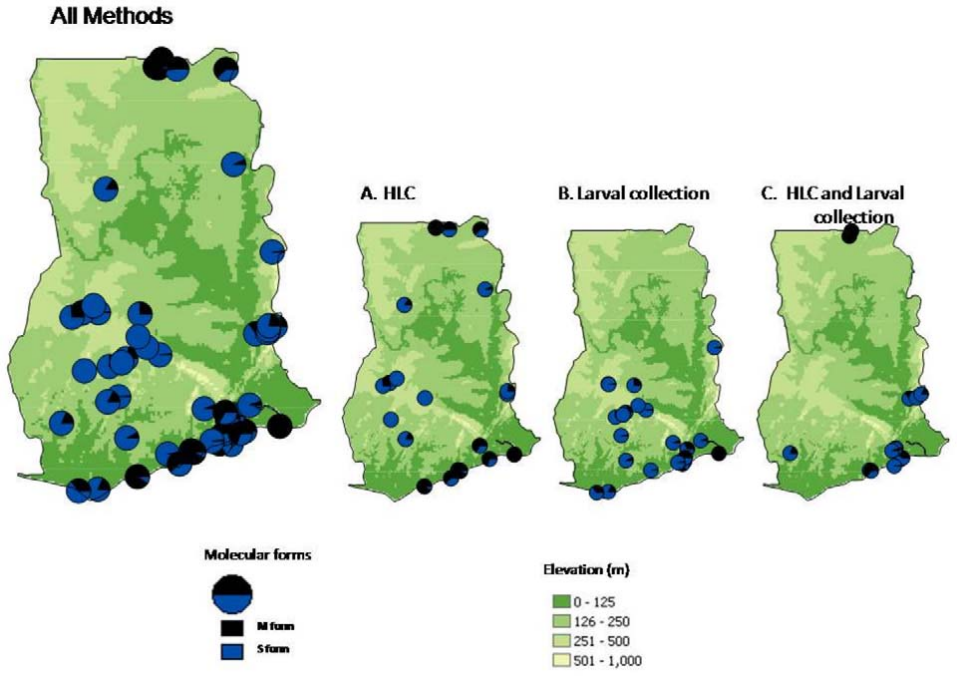
Distribution of *An. gambiae s.s* molecular form by collection methods.

Of the *An. gambiae* s.s data, a total of 70 collection sites had information on the M form (n = 2,826) and S form (n = 4,098). The collection sites were located predominately in three geographical regions i.e. the south eastern, central western and north central. The distribution of *An. gambiae* M and S forms varied across the country in different proportions (Figure 2). Figure 2 indicates the distribution of each species according to the various collection methods. Overall, *An. gambiae* M and S were sympatric in most locations. However, *An. gambiae* M form was more prevalent in the northern savanna, and coastal savanna areas of the country, and in four sites it was the only species observed. In contrast, *An. gambiae* S form was more prevalent in the middle region of the country, and in seven locations it was the only species collected. Interestingly, bivariate correlation analysis between each species indicated that their prevalences were negatively correlated (−0.763).

**Figure 3:**
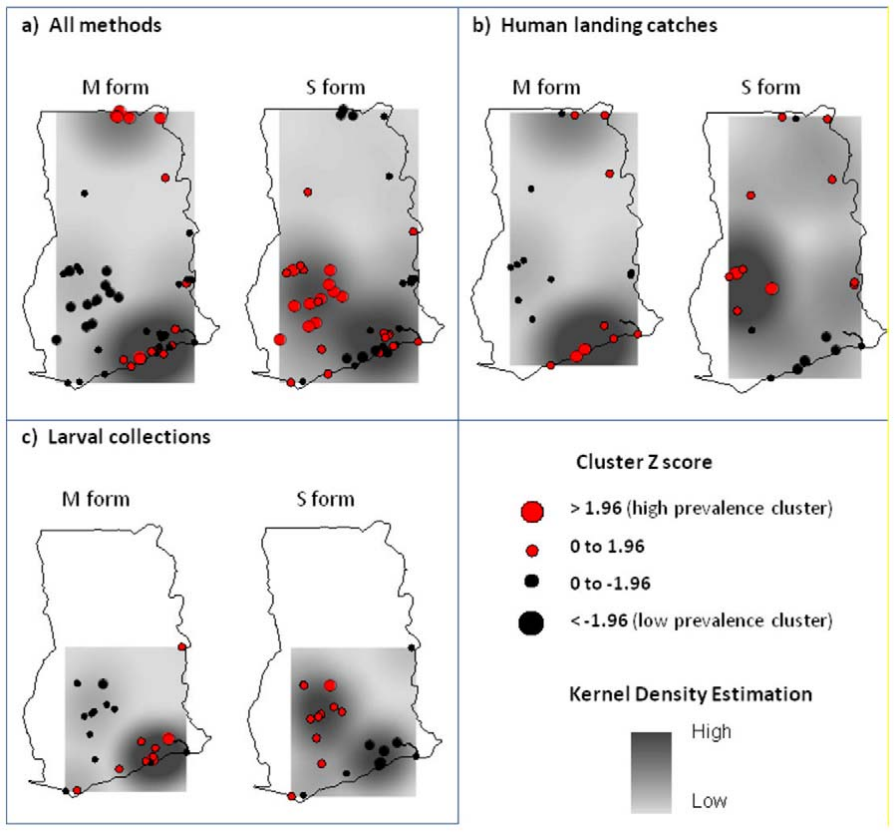
Spatial clustering trends and density distributions of *An. gambiae* s.s molecular forms.

While, it is possible that the geographical grouping of collection sites may influence trends, the spatial analyses carried out in this study indicated a positive spatial autocorrelation or clustering for both the *An. gambiae* M (MI = 0.19, Z score = 4.2, *P*≤0.01) and *An. gambiae* S (MI = 0.19, Z score = 4.2, *P*≤0.01) forms. The resultant Z scores of the Getis-Ord Gi* hot spot analyses (using inverse-distance weighting), indicated similar trends with significantly different clustering of high and low prevalences of the *An. gambiae* M and S forms. These spatial trends were overlaid a density distribution surface map shown in Figure 3, further highlighting the high to low patterns of each species.

### Environmental analysis

The relationship between the prevalence of *An. gambiae* M and S forms, and the environmental variables are shown in Table 1. Overall, bivariate correlation analysis indicated that the *An. gambiae* M form was significantly positively associated with temperature (*r* = 0.51), and negatively with elevation (*r* = −0.28), precipitation (*r* = −0.33), and humidity (*r* = −0.26). This contrasts to the *An. gambiae* S form, which was found to be significantly negatively associated with temperature (*r* = −0.58), and positively with elevation (*r* = 0.30) and rainfall (*r* = 0.41). Interestingly, elevation, precipitation and temperature correlations increased when data were stratified by the two main collection methods, HLC and larval collections (Table 1). Multiple regression analyses of all data (n = 70), indicated that temperature was an important variable for both molecular forms, explaining for *An. gambiae* M, 28% (R_2_ = 0.28, F = 25.8, P≤0.001) and for *An. gambiae* S, 36% (R_2_ = 0.36, F = 37.9, P≤0.001) of the variance in the model (Table 2).

**Table 1:** Bivariate correlations between *An. gambiae* s.s. molecular forms and environmental and epidemiological variables.

	All Methods n = 70		Human Landing Catch n = 26		Larval Collection n = 28	
	M form	S form	M form	S form	M form	S form
Elevation	−0.28*	0.30*	−0.54**	0.58**	−0.42*	0.51**
NDVI	−0.14	0.22	−0.18	0.19	−0.18	0.25
Rainfall	−0.33**	0.41**	−0.63**	0.75**	−0.39*	0.60**
Temperature	0.51**	−0.58**	0.61**	−0.72**	0.16	−0.42**
Humidity	−0.26*	0.08	−0.07	−0.10	0.19	−0.43
LF	0.46**	−0.48**	-	-	-	-
Malaria	−0.14	0.26*	-	-	-	-

*Correlation is significant at the 0.05 level (2-tailed).

**Correlation is significant at the 0.01 level (2-tailed).

**Table 2:** Multiple regression model for environmental variable predicting the presence of the *An. gambiae* M and S forms.

Species/predictor variable	Standardized Coefficient beta	T statistic	P value
*An. gambiae* M (Constant)		−4.76	<0.0001
Temperature	0.527	5.08	<0.0001
*An. gambiae* S (Constant)		6.67	<0.0001
Temperature	−0.601	−6.16	<0.0001

For each molecular form, comparisons of environmental measures between locations with significantly high and low prevalences, defined by positive Z scores (≥+1.96) and negative Z scores (≤−1.96) respectively, are shown in Table 3. Overall, locations with high *An. gambiae* M prevalences had higher NDVI and temperatures, but lower elevation, precipitation and humidity measures than those locations with lower prevalences by these species and/or where the prevalence of *An. gambiae* S form was higher. Statistical comparisons indicated significant differences (*P* value<0.004 Bonferroni corrected) between elevation, precipitation and temperature for both *An. gambiae* M and S forms between the high and low prevalence areas.

**Table 3:** Comparison of mean environmental and epidemiological measures between high and low prevalence areas of *An. gambiae* s.s molecular forms.

Variable	*An. gambiae* s.s			
	M Form	High Low	S Form	High Low
**Elevation**	147	276**	272	107**
**NDVI**	0.42	0.50	0.49	0.44
**Precipitation**	2.1	3.1**	3.1	2.0**
**Temperature**	27.0	24.9**	24.9	26.8**
**Humidity**	0.0161	0.0181	0.0181	0.0174
**LF**	20.0	2.4**	2.2	17.1**
**Malaria**	55.9	47.0	55.8	33.9

Note. High  =  Z score≥+ 1.96, Low =  Z score≤−.1.96.

**Significant at the 0.004 level after Bonferroni Correction.

**Figure 4:**
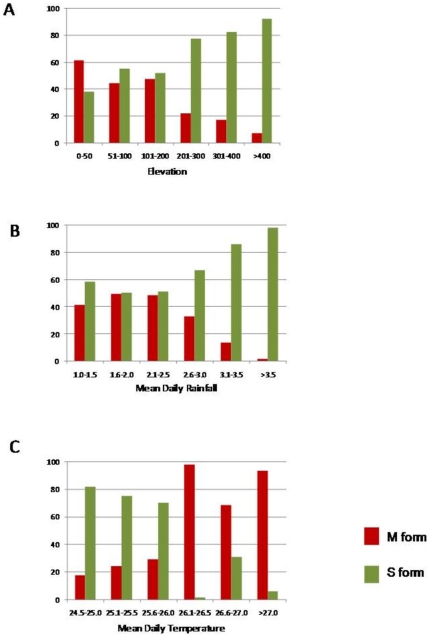
Mean prevalence of *An. gambiae* M and S forms plotted against elevation, precipitation, and temperature groupings.

To further explore the differences in elevation, precipitation and temperature, the mean prevalence of *An. gambiae* M and S forms was plotted across a range of environmental groupings (Figure 4). *An. gambiae* M prevalences were found to be higher at elevations of 0–200 m, and where mean daily precipitation ranged between 1.0–2.5 mm, and mean daily temperatures ranged between 26.1–27.6°C. In contrast, *An. gambiae* S prevalences were found to be higher at elevations >200 m, and where mean daily precipitation ranged between 2.6–3.8 mm, and mean daily temperatures ranged between 24.5–26.0°C.

### Disease association

The relationship between the *An. gambiae* M and S, and LF and malaria prevalences were first examined using bivariate correlation analysis. Results shown in Table 1, indicate that *An. gambiae* M was significantly positively associated with LF (*r* = 0.46), while *An. gambiae* S form was significantly negatively associated with LF (*r* = −0.48), but positively with malaria (*r* = 0.26). Interestingly, correlation analysis between each mosquito species, and each disease indicated significant negative associations between *An. gambiae* M and *An. gambiae* S (r = −0.76), and between LF and malaria prevalence (r = −0.41).

Second, we compared disease prevalences between high and low *An. gambiae* M and S sites (as described above). Overall, locations with high *An. gambiae* M prevalences (Z scores≥+1.96), were found to have significantly higher LF prevalences (20%) than those locations with low prevalences (Z scores≤−1.96), by these species (2.4%) and/or where the prevalence of *An. gambiae* S form was significantly high (2.2%). No significant differences were found between malaria prevalence and each mosquito species (Table 3).

**Figure 5:**
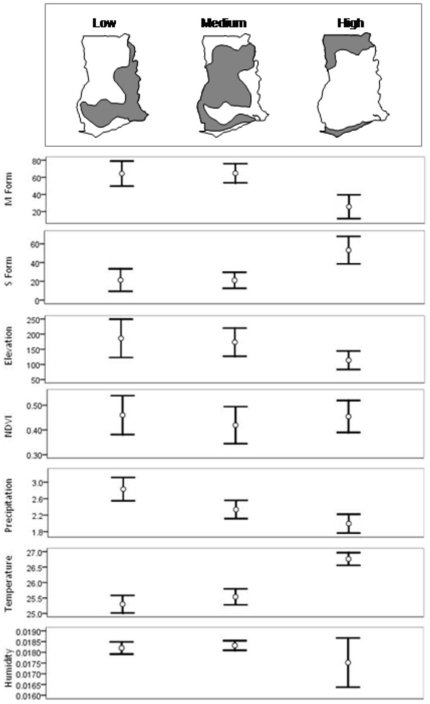
Summary of entomological and environmental variables in different LF transmission zones. Note: The maps represent the LF transmission zones in Ghana. The graphs below summarize the mean entomological and environmental measures for the sites within each zone. NDVI stands for Normalized Difference Vegetation Index.

Finally, we examined the LF data in Gyapong et al. 2002 [13], and identified three main transmission zones i.e. zero/low (<1%, n = 19), medium (1–10%, n = 32) and medium/high (10–30%, n = 19). Graphical presentation of these three zones is included in Figure 5, which summarizes mean entomological and environmental measures for the sites within each zone. In the medium/high LF transmission zone, the mean *An. gambiae* M prevalence (53.2%,) and temperatures (26.8°C) were found to be significantly higher (±2SE), and *An. gambiae* S prevalence (25.6%) significantly lower, than those found in medium transmission (21.1%; 25.5°C; 64.7%) and low transmission (21.4; 25.3°C; 64.4%) zones. In the zero/low transmission zone, precipitation measures (2.83 mm) were found to be significantly higher (+2SE), than those in the medium transmission (2.34 mm) to high transmission (1.99 mm) zones (Figure 5). Refer to the supplementary File S2 for further details on Figure 5.

## Discussion

Most entomological studies on *An. gambiae* s.l in Ghana [3], [4] have focused on small area/district based collections except the study by Yawson and colleagues [12]. Although the study by Yawson *et al.* attempted a broader coverage, this study represents the first nationwide review aimed at identifying the distribution of the various member species of the *An. gambiae* s.l, the molecular forms of the *An. gambiae* s.s, together with key environmental drivers and how they may relate to diseases. This study confirms previous observations that *An. gambiae* s.l is the major human biting mosquito species in Ghana [3], [4], [12], [26] and within the *An. gambiae* s.l, *An. gambiae* s.s is the predominant species [3], [4]. Other members of the *An. gambiae* s.l found in Ghana are *An. arabiensis* and *An. melas*.

The distribution of the *An. gambiae* M and S forms varied significantly across the country. The two molecular forms were found sympatrically in most locations, except in some areas in the middle region of the country where only the *An. gambiae* S form was observed, and in certain areas in the northern savanna and coastal savanna areas where only the *An. gambiae* M form was observed. This is confirmed by the spatial analysis and high Z score values in *An. gambiae* M/S form dominant areas. Also, the clustering remained relatively consistent irrespective of the different collection methods. Along the coast and in the northern savanna, the *An. gambiae* M form was predominant and clustered, while the S molecular form was most common and clustered in the middle belt. This positive spatial autocorrelation indicates that *An. gambiae* M/S distributions are geographically defined, and nearby areas are likely to comprise the same or similar species compositions, than those further away. The distribution of each species is also influenced by distinct and geographical related environmental factors and habitat characteristics. For example, the dominance of the *An. gambiae* M form in the northern and coastal savanna areas may be due to the wide presence of permanent breeding conditions provided by irrigation facilities [27] and ponds of water resulting from rivers run-offs since the M form is known to be associated with flooded areas, while the S form is heavily dependent on rainfall [12], [28]. The dominance of the *An. gambiae* S form in the middle region of the country may be explained by the fact that this region is mountainous, forested, with lower mean temperatures and the highest recorded rainfall in Ghana, which supports the findings of our study. In sub-Saharan Africa the abundance and distribution of *Anopheles* mosquito species is dependent on environmental factors and ecological zones [29]–[32] as well as on human population changes and anthropological effects, which may lead to land-use changes ultimately affecting vector distribution and abundance [33], [34] and as shown in a recent paper by Costantini and colleagues [35]. This current study did not focus on human factors but was able to show the wide variability in abundance and distribution of the *An. gambiae* M and S forms, which appear to be driven by a range of environmental factors. The relation between human population density and vector distribution is an indirect one and difficult to measure. However, it may be inferred that the NDVI, which is a measure of vegetation greenness and density, is somehow affected by the degree of land occupancy and exploitation by humans through the action of occupation and clearance for agricultural and developmental purposes.

Variations in the vector population densities of these two molecular forms have been observed in populations in Mali and Cameroon as well as between the various chromosomal forms in Mali [32], [35], [36]. Observations on the distributions and the predominance of the *An. gambiae* S form in a larger part of the country from this study confirm suggestions that the *An. gambiae* S form, has broader environmental ranges, and therefore is found in more locations than the M form [37]. Our environmental analyses suggest that elevation, precipitation and temperature are important variables driving the spatial distribution of each mosquito species, and the differences between them. In particular, temperature appears to be a key factor distinguishing the two species; probably due to influences on their production as reported for *An. arabiensis* and *An. gambiae s.s* [38]. The *An. gambiae* M form was more prevalent and seemingly better adapted to higher temperatures, than the S form. This is in agreement with the suggestion that the *An. gambiae* M form shows a more latitudinal range in West Africa than the S form [39], being the most dominant form encountered in hot, arid regions of the Sudan-savanna or Sahelian zones [27], [35], [40]–[42].

The mosquito vectors' association with each disease might explain the negative association between LF and malaria described by Kelly-Hope and colleagues [43]. In this current study, malaria prevalence was positively associated with the *An. gambiae* S form, whilst high LF prevalence and high transmission zones were associated with high temperatures and significantly high *An. gambiae* M prevalences. This relationship between *An. gambiae* M and S forms with LF and malaria supports previous studies that suggest the Mopti form of *An. gambiae s.s* is more associated with *W. bancrofti* than malaria transmission [44], [45] and also that it is a relatively poor vector of malaria compared with other species such as the Savanna form of *An. gambiae* [46], [47]. The LF distribution map by Gyapong and colleagues [13], indicates that the disease distribution in West Africa has the highest prevalence in the hotter Sudan/Sahel savanna areas, which are also *An. gambiae* Mopti chromosomal form dominant areas [41], [43].

The results from this study provide useful information on the distribution of the *An. gambiae* M and S forms in Ghana, highlighting the environmental factors that may play a role in determining their distributions. The information provided also marks a beginning in understanding the LF disease distribution pattern in Ghana relative to the forms of the *An. gambiae* s.s. Despite the limitations of this study in using previously modeled LF and malaria data, these results are very useful for disease control and allocation of resources, especially for LF, which together with its vectors appear to be restricted to hotter, less elevated regions of the country. Another limitation to this study is the small number of sites involved in the analysis of the *An. gambiae* M and S distribution. This coupled with their uneven distribution between the southern half and the northern half of the country could introduce potential bias in the analyses. Undertaking spatial statistical analysis with a larger dataset in the future, and further modeling their distributions through ecologic niche modeling [11], [35], [48], could further enhance our understanding of the distribution of the disease and its vectors, as well as defining the spatial limits of the vectors' distribution which is crucial for disease control. This would, however, require a constant update of the vector database generated in this study as well as collection of data from remote areas of the country where no available data exists.

## Supporting Information

File S1 Data file for Anopheles mosquito species, collection sites and coordinates, year and month of collection (where available), collection methods, numbers collected and identified, and the data sources.
(0.04 MB XLS)

File S2 Summary of entomological and environmental variables in different LF transmission zones.
(0.08 MB DOC)
