# Supplementary material for: Environmental Factors Associated with the Distribution of Anopheles gambiae s.s in Ghana; an Important Vector of Lymphatic Filariasis and Malaria
Source: PLoS One. 2010 Mar 29;5(3):e9927. doi: 10.1371/journal.pone.0009927 (PMC2847902; doi:10.1371/journal.pone.0009927)
Supplement: File S2 — Summary of entomological and environmental variables in different LF transmission zones. (0.08 MB DOC) [file pone.0009927.s002.doc]

**File S2. Summary of entomological and environmental variables in different LF transmission zones.**

|  | **Zero/Low** | **Medium** | **Medium/High** |
| --- | --- | --- | --- |
|  | **Mean (SE)** | **Mean (SE)** | **Mean (SE)** |
| **M form** | 21.4 (6.0) | 21.1 (4.3) | 53.2 (7.3)** |
| **S form** | 64.4 (7.2) | 64.7 (5.6) | 25.6 (7.0)** |
| **Elevation** | 186 (14.6) | 173 (22.7) | 113 (14.6) |
| **NDVI** | 0.46 (0.037) | 0.42 (0.037) | 0.45 (0.031) |
| **Precipitation** | 2.83 (0.13)** | 2.34 (0.11) | 1.99 (1.11) |
| **Temperature** | 25.3 (0.13) | 25.5 (0.13) | 26.8 (0.01)** |
| **Humidity** | 0.0182 (0.0001) | 0.0183 (0.0001) | 0.0175 (0.0005) |

**Note.** Comparison of the means (+ Standard Errors [SE]) indicated that there were significant differences between zones **

****** indicates that the mean measure for that zone is significantly higher than the other zones
